# Kommerell′s Diverticulum Resection With Left Brachiocephalic Artery Transfer in an Adult With Tracheoesophageal Compression: Off‐Pump Sternotomy With External Skin Cooling

**DOI:** 10.1155/crvm/6292815

**Published:** 2026-05-23

**Authors:** Michael Malyshev, Alexander Safuanov, Anton Malyshev, Dmitry Sinyukov, Andrey Rostovykh, Natalie Rostovykh, Vladlena Trushina, Alexey Malyshev

**Affiliations:** ^1^ Department of Cardiovascular Surgery, Center of Cardiac Surgery, Chelyabinsk, Russia

## Abstract

Congenital anomalies of the aortic arch often lead to tracheoesophageal compression during childhood. Occasionally, symptoms may not emerge until adulthood, appearing for the first time at any age due to age‐related or pathological dilation of the aortic segments. The oldest patient described with symptoms at the onset was 76 years old. Surgical intervention is complex and poses challenges. This case study describes a patient with a right mirror‐image branching aortic arch, Kommerell′s diverticulum, and a ligamentum arteriosum originating from the diverticulum. The patient first experienced symptoms of tracheoesophageal compression at 57 years of age. Initial treatment at another hospital, which involved division of the ligamentum and incomplete resection of the diverticulum with its fixation to the chest wall, did not alleviate symptoms. Successful off‐pump resurgery was performed through a sternotomy, which included complete resection of Kommerell′s diverticulum and direct closure of the aorta without the use of a patch. Additionally, the left brachiocephalic artery was repositioned away from the tracheoesophageal complex. External skin surface cooling to 30°C–31°C in the esophagus facilitated safe clamping of the left brachiocephalic artery during the transfer. This case demonstrates successful exposure of Kommerell′s diverticulum through sternotomy and the potential to avoid extracorporeal circulation during clamping of the brachiocephalic trunk by utilizing systemic hypothermia with external cooling.

## 1. Introduction

Congenital anomalies of the aortic arch, known as a “vascular ring,” occur when a double aortic arch surrounds the trachea and esophagus, often leading to symptoms of tracheoesophageal compression in infants and young children [1]. These anomalies make up 1.2% of congenital heart disease cases [2]. The term “vascular ring” encompasses a wide range of anomalies, with various anatomical variations depending on the development and size of the abnormal arches and branches. This variability can result in the anomaly remaining asymptomatic until adulthood, with symptoms of tracheoesophageal compression potentially arising at any age due to age‐related or pathological dilatation of the aortic arch and branches [3]. The oldest reported patient with symptoms was 76 years old [4]. Although information on the prevalence of vascular tracheobronchial and esophageal compression syndrome in adults is limited, it is believed to be very rare [2]. Before the development of tomographic imaging methods, this anomaly was frequently misdiagnosed as chronic lung diseases due to the predominance of symptoms of tracheobronchial compression. The introduction of CT and MRI has significantly improved the diagnosis process, making it noninvasive [2]. Surgical treatment is still the main option, following similar principles as in childhood. However, surgery can be difficult due to tissue rigidity, making it challenging to move surrounding structures away from the trachea and esophagus. This often requires more complex resections of the ring‐forming structures.

This case study describes a patient with a mirror‐image branching right aortic arch, Kommerell′s diverticulum, and ligamentum arteriosum arising from the diverticulum, who first experienced symptoms at 57 years old. Successful off‐pump resurgery through a median sternotomy involved complete resection of Kommerell′s diverticulum and direct closure of the aorta without a patch. Additionally, the left brachiocephalic artery was repositioned away from the tracheoesophageal complex.

## 2. Case Report

**Figure 1:**
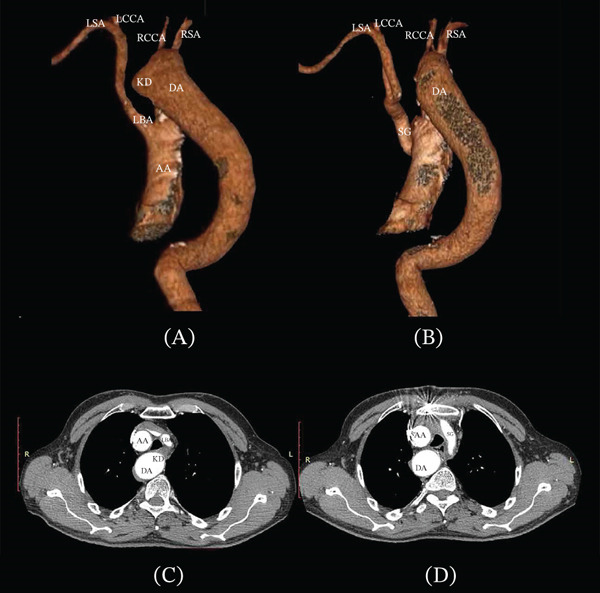
Posterior view of 3D computed tomography (CT) (A) before and (B) after the procedure. Arterial phase of the contrast‐enhanced CT scan in an axial view (C) before and (D) after the procedure. AA, ascending aorta; DA, descending aorta; ES, esophagus; IV, innominate vein; KD, Kommerell’s diverticulum; LBA, left brachiocephalic artery; LCCA, left common carotid artery; LSA, left subclavian artery; SG, synthetic graft; RCCA, right common carotid artery; RSA, right subclavian artery; TR, trachea.

**Figure 2:**
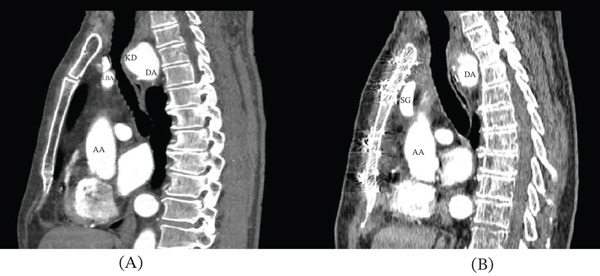
Arterial phase of the contrast‐enhanced CT scan in the left sagittal view (A) before and (B) after the procedure. AA, ascending aorta; DA, descending aorta; ES, esophagus; IV, innominate vein; KD, Kommerell’s diverticulum; LBA, left brachiocephalic artery; LCCA, left common carotid artery; LSA, left subclavian artery; SG, synthetic graft; RCCA, right common carotid artery; RSA, right subclavian artery; TR, trachea.

A 60‐year‐old male patient was admitted with symptoms of tracheoesophageal compression, including persistent coughing, difficulty swallowing, and weight loss. The patient has a long history of arterial hypertension. These symptoms first appeared when he was 57 years old. Previously, he considered himself healthy and served in the military until retirement. The esophageal symptoms were predominant. He could only consume liquid foods in small sips. Two years earlier, at another hospital, he underwent the division of the persistent ligamentum arteriosum arising from Kommerell′s diverticulum and incomplete diverticulum resection with its pexy to the chest wall in a direction away from the tracheoesophageal complex. The procedure was done via a left posterolateral thoracotomy. However, the symptoms persisted, leading to the patient being admitted for reoperation. A contrast‐enhanced chest computed tomography revealed a mirror‐image branching right aortic arch with Kommerell′s diverticulum (2.46 × 2.72 cm) and the right‐sided descending aorta. Kommerell′s diverticulum, the left brachiocephalic artery, and the aortic arch formed a compression ring around the tracheoesophageal complex (Figures 1A,C and 2A).

In this case, the size of Kommerell′s diverticulum did not exceed 3.0 cm, and all segments of the thoracic aorta were moderately dilated, with a maximum diameter of 3.8 cm. Additionally, degenerative changes in the aortic wall, such as dissection, calcification, and ulceration, were absent. Based on these findings, a decision was made to perform resection of the Kommerell′s diverticulum with simultaneous transfer of the left brachiocephalic trunk without aortic graft replacement. A decision was made to operate via a median sternotomy with the option to switch to a redo left posterolateral thoracotomy if resection of Kommerell′s diverticulum would not be possible via sternotomy. The adhesions in the pleural space further supported the median sternotomy as a surgical approach. The operation was performed with direct arterial pressure measurements in the left radial artery and the left femoral artery. Due to the risk of brain ischemia during the clamping of the left brachiocephalic artery, nonperfusion external skin surface cooling (to a temperature within the range of 30°C–31°C in the esophagus) was included in the anesthetic protocol. After setting up monitoring lines, double doses of myorelaxant were injected for suppression of shivering thermogenesis. The patient′s head, neck, front and sides of the abdomen, chest, thighs, and shanks were covered by bags containing granulated ice. When the temperature reached the target level (between 30°C and 31°C), bags were removed, except those placed on the patient′s head and neck for further temperature control. After the median sternotomy and pericardiotomy, the wide opening of the left pleural space provided excellent access to the aortic arch and Kommerell′s diverticulum. The left brachiocephalic artery was divided between two clamps. The proximal stump was sutured with a double continuous suture line, while the distal stump was anastomosed with a synthetic graft (10 mm, Vascutek Ltd.) in an end‐to‐end fashion. The opposite end of the synthetic graft was sewn into the ascending aorta with side‐biting clamping. The total time for the left brachiocephalic artery clamping was 41 min. The course of the left brachiocephalic artery was transferred to a lower position in parallel to the ascending aorta.

**Figure 3:**
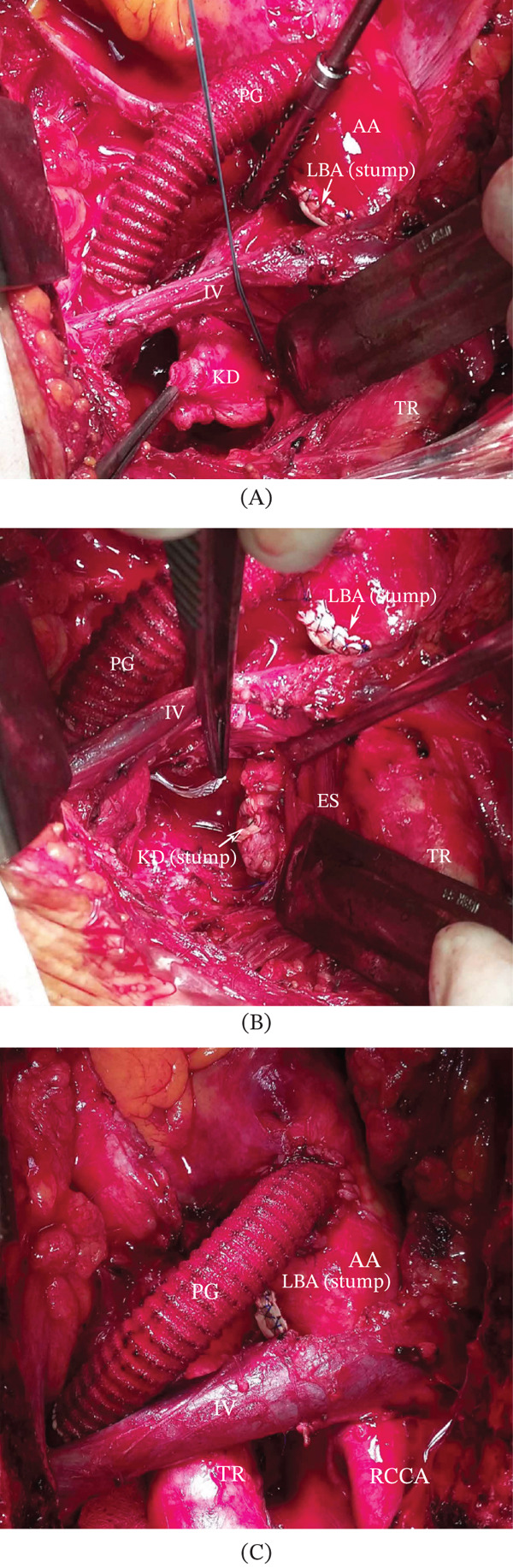
Intraoperative photos (viewed from the patient′s head) (A) before and (B, C) after Kommerell′s diverticulum resection. AA, ascending aorta; DA, descending aorta; ES, esophagus; IV, innominate vein; KD, Kommerell’s diverticulum; LBA, left brachiocephalic artery; LCCA, left common carotid artery; LSA, left subclavian artery; SG, synthetic graft; RCCA, right common carotid artery; RSA, right subclavian artery; TR, trachea.

Given the preoperative diverticulum dimensions, a side‐biting clamp was placed at the base of the diverticulum. Monitoring pressure gradients between the left radial artery and the left femoral artery allowed control of the degree of side‐biting clamping of the aorta to avoid excessive capture of the aortic lumen. The diverticulum was resected, and the aortic wall margins into the clamp were evaluated for vascular tissue strength. The aorta was then closed in the clamp with a direct suture without a patch (Figures 1B,D, 2B, and 3A–C).

Rewarming was initiated using a warming mattress and continued in the intensive care unit using a warming fan. The diverticulum tissue was sent for histopathological examination, with results interpreted as aortic wall degeneration. The patient was followed up for a 2‐year period postoperatively, and the symptoms of tracheoesophageal compression did not recur.

## 3. Discussion

Mirror‐image branching of the aortic arch and Kommerell′s diverticulum are variants of the vascular ring, typically causing symptoms of tracheoesophageal compression in neonates or children [1]. Cases that are initially asymptomatic may develop symptoms in adulthood due to age‐related or pathological aortic dilation, although this is extremely rare [4]. Due to the rarity and limited data available, operative management in adults is not well defined [5]. The long‐standing deformation of the tracheoesophageal complex leads to a less mobile anatomy of the lesion in adults compared with children, making repair much more challenging [3, 5]. Unlike children, who can undergo a pexy of the left brachiocephalic artery due to tissue compliance, adults require a more complex intervention involving clamping of the vital vessel supplying blood to the brain. Placing a side‐biting clamp at the brachiocephalic carotid artery junction can help prevent the need for complete cross‐clamping of the brachiocephalic artery. In this condition, the division of the brachiocephalic artery would be performed after creating a back‐up blood supply that would be allowed to prevent brain ischemia [4]. However, the size of the brachiocephalic artery poses a risk of excessive artery capture in the clamp, leading to brain ischemia or technical anastomosis failure. The use of nonperfusion hypothermia, achieved through skin surface cooling with a body temperature of 30°C–31°C, may be considered a simple technique to provide brain protection during brachiocephalic clamping. Technical details have been provided previously [6].

The choice of surgical approach was a particular issue in this case, as a median sternotomy was considered the best approach for transferring the left brachiocephalic artery, while a repeat left posterolateral thoracotomy was optimal for resecting Kommerell′s diverticulum [5]. In a report by Saran et al. [3], which has the largest series of operated vascular rings in adults, median sternotomy was used in only 8% of cases that did not require resection of Kommerell′s diverticulum. In this case, the diverticulum′s location, size, and aortic tissue condition allowed for free manipulation of the diverticulum through a median sternotomy. Unfortunately, transesophageal echocardiography cannot be used to assess the degree of aortic wall capture in conditions of tracheoesophageal compression due to the risk of damaging the esophagus and adjacent structures when installing a thick probe. Instead of echocardiography, measuring the pressure gradient between the left radial and femoral arteries allows for side‐biting clamping of the aorta until a slight increase (not more than 10–15 mm Hg) of the initial gradient occurs, indicating partial capture of the lumen. After diverticulum resection, aortic closure, and unclamping, the gradient returns to the initial value due to aortic wall relief. The quality of the aortic wall allowed for direct suturing of the aorta without any patches, which was considered a benefit, as a synthetic patch may lead to suture aneurysm formation due to possible ongoing aortic wall degeneration. Associated arterial hypertension may also contribute to suture line failure.

An additional advantage of median sternotomy is avoiding one‐lung ventilation, which is necessary in thoracotomy. The compression of the distal part of the trachea and bronchi complicates the installation of one‐lung ventilation [5]. Median sternotomy does not require differential intubation, simplifying anesthetic management and eliminating the risks of one‐lung anesthesia.

The borderline sizes of the aortic segments in this patient require close surveillance of the aortic dimensions for early detection of the risk of rupture or dissection.

In conclusion, mirror‐image branching right aortic arch with Kommerell′s diverticulum presenting with tracheoesophageal compression in adults is extremely rare. Combined surgery involving the transfer of the left brachiocephalic artery from the tracheoesophageal complex and resection of the diverticulum can be effectively performed off‐pump through a median sternotomy in a patient with suitable anatomy. External nonperfusion skin surface cooling is a technically simple, cost‐effective technique to provide brain protection during left brachiocephalic artery clamping. Close surveillance of aortic dimensions for early detection of the risk of rupture or dissection is recommended for adults with aortic dilatation.

## Funding

No funding was received for this manuscript.

## Consent

The patient allowed personal data processing, and informed consent was obtained.

## Conflicts of Interest

The authors declare no conflicts of interest.

## Data Availability

All data generated or analyzed during this study are included in this published article.
